# Short Physical Performance Battery and Study of Osteoporotic Fractures Index in the Exploration of Frailty Among Older People in Cameroon

**DOI:** 10.3389/ijph.2023.1605900

**Published:** 2023-08-07

**Authors:** Salvatore Metanmo, Nadine Simo-Tabue, Callixte Kuate-Tegueu, Michel Bonnet, Antoine Gbessemehlan, Fabiola Metanmo, Moustapha Dramé, Maturin Tabue-Teguo

**Affiliations:** ^1^ Inserm U1094, IRD U270, EpiMaCT—Epidemiology of Chronic Diseases in Tropical Zone, Institute of Epidemiology and Tropical Neurology, Omega Health, University of Limoges, CHU Limoges, Limoges, France; ^2^ Pôle de Gériatrie/Gérontologie CHU de Martinique, Equipe EpiCliV, Université des Antilles, Fort-de-France-Martinique, France; ^3^ Faculty of Medicine and Biomedical Sciences, The University of Yaoundé 1, Yaounde, Cameroon; ^4^ Neurology Department, Laquintinie Hospital of Douala, Douala, Cameroon; ^5^ Inserm U1219 Bordeaux Population Health Center, Université de Bordeaux, Bordeaux, France; ^6^ Division of Geriatrics, Limoges Hospital Center, Limoges, France; ^7^ Department of Clinical Research and Innovation, University Hospital of Martinique, Fort-de-France, Martinique, France; ^8^ Equipe EpiCliV, Université des Antilles, Pointe à Pitre, France

**Keywords:** frailty, Africa, elderly, epidemiology, Cameroon

## Abstract

**Objectives:** To investigate the relationship between the Short Physical Performance Battery (SPPB) and the Study of Osteoporotic Fractures (SOF) index.

**Methods:** We present data from a cross-sectional survey conducted in Cameroon. Frailty was defined as an SOF index > 0. The sensitivity and specificity of the SPPB were investigated. Principal component analysis (PCA) was performed to assess the contribution of each subtest of the SPPB to the relationship with the SOF.

**Results:** Among 403 people included (49.6% women), average age of 67.1 (±6.2) years, 35.7% were frail according to the SOF. After determining the best SPPB threshold for diagnosing frailty (threshold = 9, Se = 88.9%, Sp = 74.9%), 47.9% were frail according to the SPPB. The first dimension of PCA explained 55.8% of the variability in the data. Among the subtests of the SPPB, the chair stand test item was the component most associated with the SOF index.

**Conclusion:** Despite the overlap between the SOF and the SPPB, our results suggest that a negative result on the five chair-stands test alone would be sufficient to suspect physical frailty.

## Introduction

Frailty is a geriatric condition characterized by an increased vulnerability to external stressors [1]. This state increases risk of occurrence of adverse health conditions, such as falls, disability, dependence, nursing home admission, hospitalization, and death [2]. Many measurement tools have been developed since the 1990 to assess or measure frailty in older adults. Some of them are used in population-based studies as screening tools, while others are more suitable and effective for the screening and/or diagnosis of frailty in the clinical setting [1]. Several operational definitions of frailty exist [1, 3, 4]. The Study of Osteoporotic Fractures SOF index [5] is one of the most promising instruments for the assessment of frailty across healthcare settings, including among community-dwellers [6]. The SOF index is associated with adverse health events [6, 7]. It is easy and quick (less than 5 min) to perform, and is useful both for screening purposes in population-based studies, and for the diagnosis of frailty in the clinical setting [1]. The SOF essentially captures the physical dimension of frailty.

The Short Physical Performance Battery (SPPB) first described in 1994 [8] is a widely used test for measuring functional status and physical performance [8–12], particularly lower limb function. It is a composite comprising three sub-tests, namely, the balance test, the gait speed test and the chair stand test. As with the SOF index, low SPPB scores are predictive of various health events, including falls, limitations in the activities of daily living, disability, admission and readmission to hospital [13]. A recent review reported an association between lower SPPB scores and higher mortality risk [12]. The SPPB is also used in the assessment of sarcopenia, especially in the community context [14–16].

In Africa, few studies have used the SPPB to assess physical frailty, even though it is now well established that physical frailty is a geriatric syndrome whose main cause is sarcopenia, a pathology linked to loss of muscle mass and strength [11, 17–20]. To the best of our knowledge, no study in Africa has evaluated the SPPB compared to the SOF. Despite the likely risk of overlap, we feel it is important to determine the relationship between SPPB and SOF, and to identify which component of SPPB is most associated with frailty (as assessed by the SOF) in clinical practice among persons aged over 55 years in Cameroon. Considering that the component of the SPPB contribute to the total score of the SPPB, each component probably has a different weight, especially in clinical practice. Determining the contribution of each component could help clinicians to better identify the frailty syndrome. Similarly, a component with a high weighting could also be leveraged to improve knowledge among patients and their families, in order to propose pre-diagnosis at home.

The aim of this study was to explore the relationship between the SOF index and the SPPB (accuracy diagnostic of SPPB) as a screening tool for physical frailty in a population of older people from sub-Saharan Africa (SSA), and secondly, to identify which component of the SPPB is most associated with the SOF index.

## Methods

Between 1st January and 31st May 2019, a cross-sectional study was conducted among the general population in the city of Douala in Cameroon. Any person aged 55 years and over who was a member of a mutual health insurance company (MUPAC), able to stand up without support, and able to walk 4 m was eligible. Participants with severe health problems, including neurological disorders and blindness were excluded. The details of the survey procedures have previously been described elsewhere [16].

### Assessment of SPPB

The SPPB is a battery comprising three tests, each scored from 0 to 4 [8]. The first is the gait speed test, which assesses the time taken to walk 4 m. The second is the chair stand test, in which the participant is asked to perform five chair-stands as quickly as possible, without using the arms, and the time taken to do the five chair-stands is recorded. The last component of the SPPB is the balance test, in which the participant’s ability to stand with their feet in each of three positions (side-by-side stand, semi-tandem stand and tandem stand) is assessed. Each position must be maintained for at least 10 s. The less time required, the better the physical performance. The overall score (SPPB) also follows the same interpretation rule.

### Assessment of Frailty Status: The SOF Index

Frailty status was assessed using the SOF index [21], which comprises three items, each scored from 0 to 3. The first is self-reported involuntary weight loss—the participant meets the criterion for weight loss if they lost >5% of their body weight in last 2–3 years without intent to lose weight. The second item is a chair stand test, which assess the participant’s ability to get up from a chair 5 times without using their arms. The participant meets the chair-stand criterion is they fail to get up successfully all 5 times. The third items is reduced energy level, corresponding to a participant who replies “No” to the question “Do you feel full of energy?”. Higher SOF scores correspond to greater frailty. For the purposes of this study, participants with an SOF index ≥1 were considered frail.

This study was approved by the Ethics Committee of the Université des Montagnes (Bangangté-Cameroon) under the number N°2019/049/UdM/CIE. Written informed consent was obtained from all participants before inclusion in the study.

The full procedures for performing and scoring the SPPB and SOF index are given in the Supplementary Files S1, S2. All participants performed both tests. However, the SPPB and the SOF index were performed at a distance (2 days) from each other in order to avoid bias in the results due to test repetition.

### Statistical Analysis

Descriptive analysis of the socio-demographic and clinical characteristics of the study population was performed. Quantitative data are presented as medians and interquartiles and categorical variables are presented as number and percentage. To explore the relationship between the SPPB and the SOF index, several analyses were conducted. First, we calculated the correlation coefficient between the SOF index and the SPPB, and between the SOF index and each sub-test of the SPPB. Second, to plot a Receiver Operating Characteristic (ROC) curve between the SPPB and the SOF index, several different thresholds of the SPPB were tested. For each threshold tested, the rate of true and false positives in relation to the SOF index was recorded. The area under the ROC curve was estimated (AUC). Third, based on the most discriminant threshold of the SPPB, we calculated the Kappa coefficient for agreement between the SPPB and the SOF (both considered as categorical variables) with the associated confidence interval. The Landis and Koch classification [22] was used to characterize agreement as poor, slight, fair, moderate, substantial or almost perfect.

Principal component analysis (PCA) was performed between the subtests of the SPPB and the SOF. Active variables used to perform PCA were: balance test, gait speed test, chair stand test and SOF frailty index. The overall SPPB score, age and sex were used as supplementary observations. PCA was performed after data were centered or normalized. A study of the variables and individuals was carried out after PCA had been performed. *p*-values < 0.05 were considered statistically significant. All analyses were performed using the software R version 4.0.3.

## Results

### Study Population Characteristics

The median age of the study population was 67 years, and 49.6% were female. The median score of the different subtests of the SPPB was 3, while the median total SPPB score was 10.0 (8.0, 11.0). The median SOF score was 0.0 (0.0, 1.0) and 144 (35.7%) participants had a score ≥ 1, and were considered to have physical frailty. The other socio-demographic and clinical characteristics of our population are presented in Supplementary Table S1.

### Relationship Between SPPB and SOF

#### Correlation Between the Two Methods

The Spearman correlation coefficients for the correlations between the SPPB, its subtests, and the SOF index are presented in Table 1. The SOF and SPPB were strongly negatively correlated (*r* = −0.68). The three components of the SPPB were significantly negatively correlated with the SOF index, but of these three components, only the correlation between the SOF index and the chair stand test (−0.67) was as strong as the correlation between the SOF and the SPPB.

**TABLE 1:** Correlation between the study of osteoporotic fractures index and the subtests of the short physical performance battery, Douala, Cameroon. 2019.

Variables	Spearman’s correlation coefficient	*p*-value
Total SPPB	−0.68	<0.001
Balance test	−0.33	<0.001
Gait speed test	−0.39	<0.001
Chair stand test	−0.67	<0.001

SPPB, short physical performance battery.

#### ROC Curve and AUC

**FIGURE 1:**
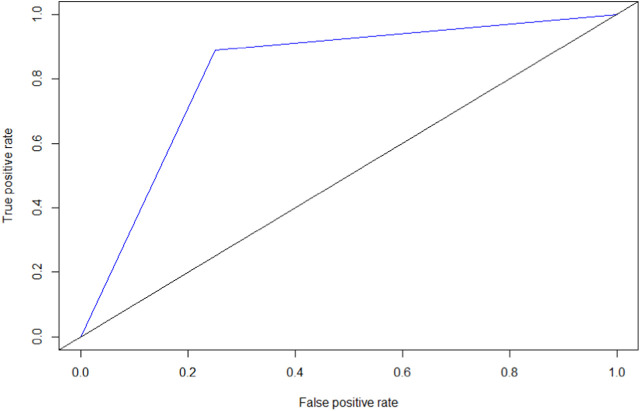
Receiver operating characteristic curve for the short physical performance battery and the study of osteoporotic fractures index, Douala, Cameroon. 2019.

Different thresholds for the SPPB were tested, and the resulting ROC curve for the relation between the SPPB and SOF is shown in Figure 1. A selection of thresholds with their associated diagnostic performance is presented in Supplementary Table S2. An SPPB score of 9 was the cut-off that best discriminated impaired from non-impaired participants (Youden index = 0.64). The area under the ROC curve (AUC) was 0.82.

#### Agreement Between the Two Methods

To calculate the agreement between the SPPB and the SOF index, the threshold of 9 was used, as per the previous analysis; 193 (47.9%) participants had an SPPB score ≤ 9. Table 2 shows the distribution of patients according to each of the two assessment methods (SPPB and SOF). The Kappa coefficient was 0.60 [95% CI: 0.52–0.67], *p* < 0.001, corresponding to moderate agreement between the SPPB and the SOF for frailty screening.

**TABLE 2:** Agreement between classification on the short physical performance battery (using a cut-off = 9) and the study of osteoporotic fractures index, Douala, Cameroon. 2019.

		SOF		Total
		Frail	Robust	
SPPB	Frail	128	65	193
	Robust	16	194	210
	Total	144	259	406

SOF, study of osteoporotic fractures index; SPPB, short physical performance battery.

#### Principal Component Analysis (PCA)

##### Description of the Dimensions

Supplementary Figure S1 shows the different dimensions derived from the PCA and the percentage of inertia. The first two dimensions explained 77% of the total variability, i.e., 77% of the information in the data was summarized by the first plane of the PCA. Note that a plane is made up of two consecutive dimensions. Thus, dimension 1 and dimension 2 form the first plane, while dimension 3 and 4 form the second plane, and so on.

##### Study of Variables

**FIGURE 2:**
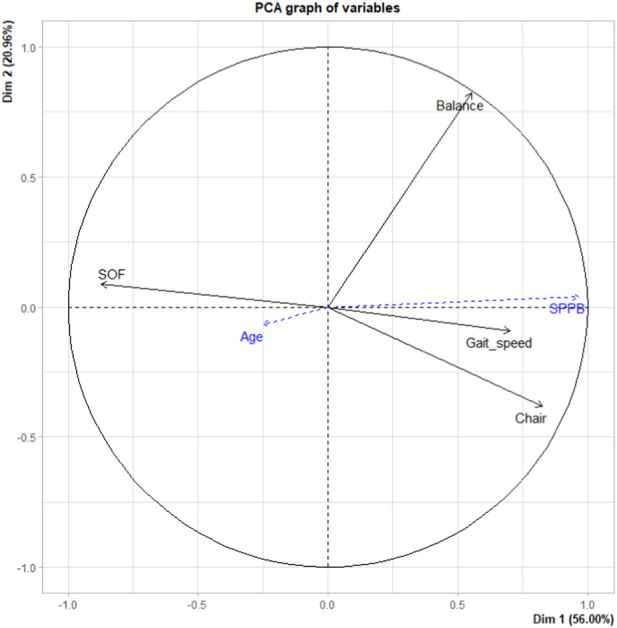
Representation of variables in the first plane by principal component analysis, Douala, Cameroon. 2019.

All variables were associated with dimension 1. The correlation coefficient was greater than |0.5| for all, except age (correlation = −0.248) (Supplementary Table S3). From Supplementary Table S3 and Figure 2, it can be seen that the subtests of the SPPB, as well as the overall SPPB score were positively correlated with the first dimension, while the SOF index was negatively correlated with this dimension. From Figure 2, it can be seen that the SOF index, the balance test, the chair stand test and the SPPB are well represented by the PCA in the first plane, unlike age, which is not well represented.

Second plane (dimensions 3 and 4): Gait speed was the best represented variable on the second plane, mainly by the third dimension (Supplementary Figure S2), with which it was positively correlated (correlation coefficient = 0.71).

###### Quality of Projection of Variables in the Plane

Apart from age, all variables were well represented in the first plane. Table 3 shows the quality of representation (Cos2) of each variable on the first two dimensions and therefore in the foreground. The balance test is the best represented variable in the main PCA plane, in particular by the second dimension. The chair stand test, the overall SOF index and the SPPB were mostly represented by the main dimension of the PCA.

**TABLE 3:** Quality of projection and contribution of the variables to the construction of the first plane, Douala, Cameroun. 2019.

Variables	Cos2			Contribution	
	Active variables				
	Dimension 1	Dimension 2	1st plane	Dimension 1	Dimension 2
Balance test	0.306	0.677	0.983	13.649	80.707
Gait speed test	0.492	0.008	0.5	21.951	0.993
Chair stand test	0.681	0.146	0.827	30.382	17.402
SOF	0.762	0.008	0.77	34.018	0.898
	Supplementary variables				
SPPB	0.932	0.002	0.934		
Age	0.062	0.004	0.066		

SOF, study of osteoporotic fractures index; SPPB, short physical performance battery.

###### Contribution of the Variables to the First and Second Main Dimensions

The main contributor to the creation of dimension 1 was the SOF, although its contribution was not very large (ctr = 34.018), followed by the chair stand test (ctr = 30.382). The contributions of the other variables to the creation of the different PCA dimensions are presented in Table 3.

###### Qualitative Variable: Sex

**FIGURE 3:**
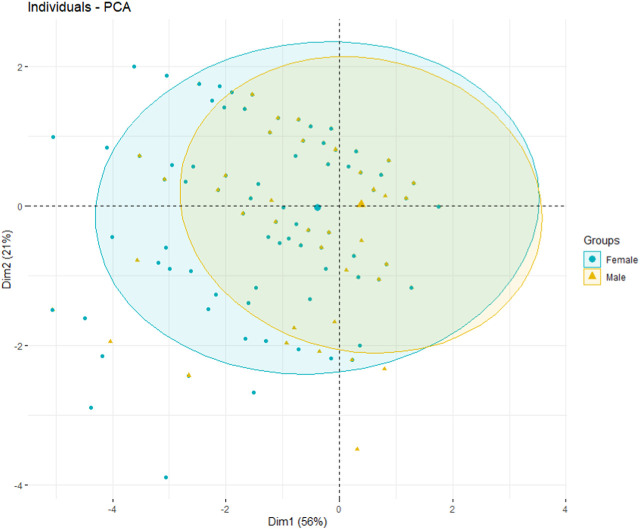
Graph of individuals grouped by sex, Douala, Cameroon. 2019.

About 6.7% (correlation ratio *R* ^2^ = 0.067) of the variability of the coordinates of individuals on the first dimension was explained by the variable “sex”. The barycenter of men is located on the right of the graph, while that of women is on the left (Figure 3). In other words, compared to women, men more often had a higher score on the SPPB and its components. Supplementary Table S4 presents the quality of presentation of each modality of the sex variable, as well as the associated v.tests. The v.test values indicate that on dimension 1, the coordinates of the sex category are significantly different from zero.

##### Study of Individuals

The SPPB, gait speed test, balance test and chair stand test variables were all positively related to dimension 1. Individuals on the right side of the graph had high values for these tests, while individuals on the left had lower scores. Conversely, individuals on the left of the graph had a high SOF index while those on the right had a lower SOF score (Figure 3).

Dimension 2 was highly correlated with the balance test (*r* = 0.82). Individuals on the upper part of the graph had higher scores on the balance test while those on the lower part of the graph had lower scores on this test (Figure 3).

Supplementary Figure S3 displays the individuals graph, showing a selection of individuals whose representation quality is > 0.8. The qualitative variable “sex” is also represented by the barycenters for each sex (male/female). Figure 3 shows the graph of individuals with ellipses. Individuals are colored by sex. The ellipse for men is shifted slightly to the right compared to that of women, which supports the fact that men often had a higher overall score on the SPPB than women, and conversely a lower SOF score than women. This slight difference between women and men is only significant on dimension 1 of the PCA due to the value of the v.test.

## Discussion

### Prevalence of Frailty and the Value of the SOF Index as a Screening Tool

According to WHO and the World Bank, despite being on an upward trend, life expectancy in Cameroon was only 58 years at birth in 2019 [23, 24]. This low value justifies why in Cameroon, as in other SSA countries, people are considered to be “old” relatively earlier than their Caucasian counterparts. The median age (67 years) found in our sample is therefore not exceptional in this part of the world and could be representative of older people in Cameroon. The prevalence of physical frailty assessed using the SOF index (35.7%), despite our selection criteria, could be an argument in favor of this representativeness. The SOF index is a validated tool in the assessment of physical frailty and is widely used in the literature in many fields of application [6, 25–27]. One diagnostic study reported that the SOF index was of good value for diagnosing frailty (specificity = 99.5%); it is therefore more valuable as a diagnostic tool than as a screening tool [28].

### The Relationship Between SOF Index and SPPB

The best SPPB cut-off for assessing frailty in this population was nine points. Indeed, this cut-off had the best Youden index (0.64), and an AUC of 0.82, for sensitivity and specificity of 88.9% and 74.9%, respectively. Our results are in agreement with several studies that have used a cut-off of 9 in the SPPB to differentiate frail from robust people [18, 29, 30]. However, other cut-off values can be found depending on purpose of performing the SPPB. For example, one study reported that an SPPB score ≤8 was the best cut-off when the objective was to determine frailty, while the optimal threshold was ≤10 when the objective was to determine onset of the frailty process (pre-frailty) [20]. Another recent study found that using a threshold of ≤7, sensitivity and specificity were very similar (0.80 and 0.83, respectively) [17]. One of the common reasons for these differences in results could be the wide variability of reference tests in the diagnosis of frailty. Indeed, in this study, frailty was determined using the Cardiovascular Health Study (CHS) phenotype (phenotype model), 26- and 34-item frailty index (deficit accumulation model) [17]. Their survey was conducted in a general population (like ours) and the gender distribution was similar (54.8% were female in their study) [17]. Conversely, the higher age (76 ± 6.8 years) in their population could explain the discrepancies between our two populations.

In addition to the reference test, the difference between our results and other reports in the literature may be due to other factors such as socio-economic level. For example, in a multicenter diagnostic study evaluating the physical performance of the SPPB (with a threshold of 9) in the diagnosis of frailty using the Fried phenotype (i.e., weight loss, exhaustion, weakness, mobility limitation and low physical activity) as a reference, the SPPB had better diagnostic performance (Se = 92%, Sp = 80%) in a population in Canada and worse diagnostic performance (Se = 81%, Sp = 52%) in a population in Brazil [18]. The authors underlined that the SPPB discriminated frailty better among older people with a higher socio-economic level.

The correlations found between the SPPB (and its subtests) and the SOF index remain mathematical and require careful interpretation. A measure of agreement using the Kappa index is more clinically relevant. The agreement between the SPPB and the SOF index was moderate (according to the Landis and Koch classification) and significant for a threshold ≤9 on the SPPB. The ability of the SPPB to classify participants as frail or robust is therefore not a matter of chance. To the best of our knowledge, few studies in the literature have explored the agreement between the SPPB and diagnostic tools for frailty. Our study therefore provides the first results of this agreement in a population from SSA.

The majority of studies that have assessed the relevance of using the SPPB in frailty have been diagnostic accuracy studies, limited to the assessment of the diagnostic performance of the SPPB [17–20, 31]. By using PCA, our study not only enables us to explore this relationship from another point of view, but also provides additional insights. Although the total SPPB score was not an active variable in the PCA, the first dimension of the PCA appeared to be the SPPB, with a very strong correlation (0.96) observed between the SPPB and the first dimension. In other words, studying the different correlations between the variables and the first dimension (which is the main dimension of the PCA) amounts to studying the correlations between these variables and the total score of the SPPB.

### The SPPB and the SOF Index Overlap

Indeed, both these tests contain the chair stand test item. The objective of this sub-test is different in the SPPB, which measures the time taken to do the five chair-stands, as compared to the SOF, where the ability to do the five chair-stands is assessed in a binary (yes/no) manner, regardless of the time taken. However, both assessment methods involve stimulation of the osteo-motor system.

As expected in our study, the subtest of the SPPB that was most strongly correlated with the first dimension was the chair stand test, and the SOF index was highly correlated with this same dimension. However, this observation provides new information beyond the simple overlap that exists between the SOF and the SPPB. Indeed, this result suggests that in the assessment of physical frailty, the chair stand test is of high value in this population, although our study population was younger. The PCA was used to synthesize the information from our 403 patients and dimension 1 had the largest share in this synthesis. Dimension 1 almost represents the SPPB, while the SOF and the chair stand test (as a subtest of the SPPB) are highly correlated with it. In other words, the information coming from these 403 individuals is best explained (or summarized) by the chair stand test. A low score on this test (regardless of the context in which it is performed) may be an early warning of physical frailty in a young-aged patient. Our study is not the first to make this observation or to conclude that the SPPB is useful for the early detection of frailty [10, 18]. Indeed, in a previous study, a low SPPB score was observed in patients with normal gait speed and according to the authors, their results suggested that the physical performance battery may detect early signs of frailty even before the onset of gait slowing [10].

The gait speed test is considered to be a good predictor of health status in the elderly. Indeed, one study showed that usual gait speed of less than 1 m/s identifies persons at high risk of health-related outcomes in well-functioning older people [32]. Another study found that gait speed alone was useful in estimating the risk of disability in community-based populations [9]. These results are different from what was observed in our population, where the test most correlated with frailty was the chair stand test. The populations in the previous studies were older than ours, and it has been suggested that walking speed impairments may occur late in the disability process [10]. Furthermore, the effect of age in the disability process is well established. We therefore hypothesize that the chair stand test has more diagnostic value in the early identification (i.e., in the young-older person who is still walking normally) of the disability process.

In short, the chair stand test alone could be proposed to clinicians in routine practice as a means to detect the onset of physical frailty in individuals from the age of 55. This test is simple to perform, and can also be proposed to patients’ families for even earlier screening. Like Cameroon, sub-Saharan African countries (or more generally developing countries) face a number of challenges, such as lower life expectancy (lower than in developed countries), less well developed healthcare systems (fewer efficient screening tools and healthcare personnel, for example), and sometimes precarious sanitary conditions. The chair stand test is simple to implement, and can be carried out by any general practitioner. It is not very time-consuming, and suitable for use as part of a routine consultation. It is inexpensive, requiring only a chair, and can be used for the early detection of frailty. The chair stand test is therefore an excellent alternative for the screening and exploration of frailty in these countries.

A more recent study performed PCA only with the three subtests of the SPPB, to investigate the contribution of each subtest to the overall score [11]. The authors found that all sub-tests contributed more or less equally to the overall score, with a slightly greater contribution from the balance test. These results cannot really be considered different from ours because of the active variables included in the PCA. Indeed, our PCA was constructed on four scores including the SOF index score and therefore the information is certainly on the subtests of the SPPB but related to frailty. In this PCA, moreover, the balance test is better represented, more strongly correlated, and contributed above all to the construction of the second dimension. It therefore probably measures something else in this population.

When considering frailty in the elderly, the notions of age and gender are two determining factors [33, 34]. The PCA also explored the role of gender in frailty. In our population, men appeared to have better physical performance than women, particularly with regard to the chair stand test, although this difference seems minimal.

Our study has some limitations. First, it was a cross-sectional study and the results deserve to be consolidated by longitudinal studies. The choice of the reference test (SOF index), which is known to overlap with the SPPB, could also be considered as a limitation. However, this choice enabled us to highlight the important role of the subtest at the origin of this overlap. Finally, we do not have the scores for each sub-test of the SOF index, as we did for the SPPB. This would have enabled us to better explore these two tests, particularly to observe differences and the impact that the two methods of doing the chair stand test may have. Conversely, some strengths of our study can be noted. First, this is the first time that a study of this type has been conducted in a population from SSA, and sample size is relatively large. Second, exploring the relationship between frailty and SPPB test scores via PCA enabled a different approach to the value of each subtest in screening/diagnosing frailty.

### Conclusion

In conclusion, the SPPB could be a valid tool for the identification of frailty in older people in SSA. Our results suggest that in a young geriatric population, a low score on the chair stand test could be an early warning sign of failing health, even when the scores of the other subtests and the overall SPPB score appear to be good. This test alone could be offered both in routine clinical practice and to families for early detection of frailty. However, these hypotheses need to be tested through longitudinal studies.

## References

[1] Dent E Kowal P Hoogendijk EO. Frailty Measurement in Research and Clinical Practice: A Review. Eur J Intern Med (2016) 31:3–10. 10.1016/j.ejim.2016.03.007 27039014

[2] Fried LP Tangen CM Walston J Newman AB Hirsch C Gottdiener J Frailty in Older Adults: Evidence for a Phenotype. J Gerontol A Biol Sci Med Sci (2001) 56(3):M146–156. 10.1093/gerona/56.3.m146 11253156

[3] Checa-López M Oviedo-Briones M Pardo-Gómez A Gonzales-Turín J Guevara-Guevara T Carnicero JA FRAILTOOLS Study Protocol: A Comprehensive Validation of Frailty Assessment Tools to Screen and Diagnose Frailty in Different Clinical and Social Settings and to Provide Instruments for Integrated Care in Older Adults. BMC Geriatr (2019) 19(1):86. 10.1186/s12877-019-1042-1 30885132 PMC6423863

[4] Redín-Sagredo MJ Aldaz Herce P Casas Herrero A Gutiérrez-Valencia M Martínez-Velilla N. Heterogeneity Amongst Different Diagnostic Tools in Frailty Screening. An Sist Sanit Navar (2019) 42(2):169–78. 10.23938/ASSN.0642 31322141

[5] Black DM Steinbuch M Palermo L Dargent-Molina P Lindsay R Hoseyni MS An Assessment Tool for Predicting Fracture Risk in Postmenopausal Women. Osteoporos Int J Establ Result Coop Eur Found Osteoporos Natl Osteoporos Found USA (2001) 12(7):519–28. 10.1007/s001980170072 11527048

[6] De Buyser SL Petrovic M Taes YE Toye KRC Kaufman JM Lapauw B Validation of the FNIH Sarcopenia Criteria and SOF Frailty index as Predictors of Long-Term Mortality in Ambulatory Older Men. Age Ageing (2016) 45(5):602–8. 10.1093/ageing/afw071 27126327

[7] Luciani A Dottorini L Battisti N Bertuzzi C Caldiera S Floriani I Screening Elderly Cancer Patients for Disabilities: Evaluation of Study of Osteoporotic Fractures (SOF) index and Comprehensive Geriatric Assessment (CGA). Ann Oncol Off J Eur Soc Med Oncol (2013) 24(2):469–74. 10.1093/annonc/mds471 23041592

[8] Guralnik J Simonsick E Ferrucci L Glynn RJ Berkman LF Blazer DG A Short Physical Performance Battery Assessing Lower Extremity Function: Association with Self-Reported Disability and Prediction of Mortality and Nursing home Admission. J Gerontol Medical Sci (1994) 42(2):85–94. 10.1093/geronj/49.2.m85 8126356

[9] Guralnik JM Ferrucci L Pieper CF Leveille SG Markides KS Ostir GV Lower Extremity Function and Subsequent Disability: Consistency across Studies, Predictive Models, and Value of Gait Speed Alone Compared with the Short Physical Performance Battery. J Gerontol A Biol Sci Med Sci (2000) 55(4):M221–31. 10.1093/gerona/55.4.m221 10811152 PMC12149745

[10] Verghese J Xue X. Identifying Frailty in High Functioning Older Adults with normal Mobility. Age Ageing (2010) 39(3):382–5. 10.1093/ageing/afp226 20051607 PMC2899862

[11] Tabue-Teguo M Dartigues JF Simo N Kuate-Tegueu C Vellas B Cesari M. Physical Status and Frailty index in Nursing home Residents: Results from the INCUR Study. Arch Gerontol Geriatr (2018) 74:72–6. 10.1016/j.archger.2017.10.005 29040887

[12] De Fátima Ribeiro Silva C Ohara DG Matos AP Pinto ACPN Pegorari MS. Short Physical Performance Battery as a Measure of Physical Performance and Mortality Predictor in Older Adults: A Comprehensive Literature Review. Int J Environ Res Public Health (2021) 18(20):10612. 10.3390/ijerph182010612 34682359 PMC8535355

[13] Treacy D Hassett L. The Short Physical Performance Battery. J Physiother (2018) 64(1):61. 10.1016/j.jphys.2017.04.002 28645532

[14] Jyväkorpi SK Ramel A Strandberg TE Piotrowicz K Błaszczyk-Bębenek E Urtamo A The Sarcopenia and Physical Frailty in Older People: Multi-Component Treatment Strategies (SPRINTT) Project: Description and Feasibility of a Nutrition Intervention in Community-Dwelling Older Europeans. Eur Geriatr Med (2021) 12(2):303–12. 10.1007/s41999-020-00438-4 33583000 PMC7990826

[15] Lee SY Choo PL Pang BWJ Lau LK Jabbar KA Seah WT SPPB Reference Values and Performance in Assessing Sarcopenia in Community-Dwelling Singaporeans - Yishun Study. BMC Geriatr (2021) 21(1):213. 10.1186/s12877-021-02147-4 33781211 PMC8008740

[16] Metanmo S Kuate-Tegueu C Gbessemehlan A Dartigues JF Ntsama MJ Nguegang Yonta L Self-Reported Visual Impairment and Sarcopenia Among Older People in Cameroon. Sci Rep (2022) 12(1):17694. 10.1038/s41598-022-22563-9 36271132 PMC9586958

[17] Jung HW Baek JY Jang IY Guralnik JM Rockwood K Lee E Short Physical Performance Battery as a Crosswalk between Frailty Phenotype and Deficit Accumulation Frailty Index. J Gerontol A Biol Sci Med Sci (2021) 76(12):2249–55. 10.1093/gerona/glab087 33780526 PMC8598989

[18] Da Câmara SMA Alvarado BE Guralnik JM Guerra RO Maciel ÁCC. Using the Short Physical Performance Battery to Screen for Frailty in Young-Old Adults with Distinct Socioeconomic Conditions. Geriatr Gerontol Int (2013) 13(2):421–8. 10.1111/j.1447-0594.2012.00920.x 22882512

[19] Chang SF Yang RS Lin TC Chiu SC Chen ML Lee HC. The Discrimination of Using the Short Physical Performance Battery to Screen Frailty for Community-Dwelling Elderly People. J Nurs Scholarsh Off Publ Sigma Theta Tau Int Honor Soc Nurs (2014) 46(3):207–15. 10.1111/jnu.12068 24502621

[20] Perracini MR Mello M de Oliveira Máximo R Bilton TL Ferriolli E Lustosa LP Diagnostic Accuracy of the Short Physical Performance Battery for Detecting Frailty in Older People. Phys Ther (2020) 100(1):90–8. 10.1093/ptj/pzz154 31612228

[21] Ensrud KE Ewing SK Taylor BC Fink HA Cawthon PM Stone KL Comparison of 2 Frailty Indexes for Prediction of Falls, Disability, Fractures, and Death in Older Women. Arch Intern Med (2008) 168(4):382–9. 10.1001/archinternmed.2007.113 18299493

[22] Landis JR Koch GG. The Measurement of Observer Agreement for Categorical Data. Biometrics (1977) 33(1):159–74. 10.2307/2529310 843571

[23] Organisation mondiale de la Selon. l’espérance de vie au Cameroun est inférieure à la moyenne en Afrique malgré les dépenses en santé (2022). Available at: https://actucameroun.com/2020/06/24/selon-loms-lesperance-de-vie-au-cameroun-est-inferieure-a-la-moyenne-en-afrique-malgre-les-depenses-en-sante/ (Accessed April 4, 2022).

[24] Organisation mondiale de la Selon. Espérance de vie à la naissance, hommes (années) - Cameroon | Data (2022). Available from: https://donnees.banquemondiale.org/indicator/SP.DYN.LE00.MA.IN?locations=CM (Accessed April 4, 2022).

[25] Trombetti A Hars M Hsu FC Reid KF Church TS Gill TM Effect of Physical Activity on Frailty: Secondary Analysis of a Randomized Controlled Trial. Ann Intern Med (2018) 168(5):309–16. 10.7326/M16-2011 29310138 PMC5898617

[26] Boulos C Salameh P Barberger-Gateau P. Malnutrition and Frailty in Community Dwelling Older Adults Living in a Rural Setting. Clin Nutr Edinb Scotl (2016) 35(1):138–43. 10.1016/j.clnu.2015.01.008 25649256

[27] Mohamad MI Khater MS. Evaluation of Insulin like Growth Factor-1 (IGF-1) Level and its Impact on Muscle and Bone mineral Density in Frail Elderly Male. Arch Gerontol Geriatr (2015) 60(1):124–7. 10.1016/j.archger.2014.08.011 25240725

[28] Seto E Setiati S Laksmi PW Tamin TZ. Diagnostic Test of a Scoring System for Frailty Syndrome in the Elderly According to Cardiovascular Health Study, Study of Osteoporotic Fracture and Comprehensive Geriatric Assessment Based Frailty Index Compared with Frailty Index 40 Items. Acta Med Indones (2015) 47(3):183–7. 26586383

[29] Imamura K Yamamoto S Suzuki Y Yoshikoshi S Harada M Osada S Comparison of the Association between Six Different Frailty Scales and Clinical Events in Patients on Hemodialysis. Nephrol Dial Transpl Off Publ Eur Dial Transpl Assoc - Eur Ren Assoc. (2022) 38:455–62. 10.1093/ndt/gfac047 35212731

[30] Bandinelli S Lauretani F Boscherini V Gandi F Pozzi M Corsi AM A Randomized, Controlled Trial of Disability Prevention in Frail Older Patients Screened in Primary Care: The FRASI Study. Design and Baseline Evaluation. Aging Clin Exp Res (2006) 18(5):359–66. 10.1007/BF03324831 17167299 PMC2659809

[31] Kim MJ Yabushita N Kim MK Nemoto M Seino S Tanaka K. Mobility Performance Tests for Discriminating High Risk of Frailty in Community-Dwelling Older Women. Arch Gerontol Geriatr (2010) 51(2):192–8. 10.1016/j.archger.2009.10.007 19939477

[32] Cesari M Kritchevsky SB Penninx BWHJ Nicklas BJ Simonsick EM Newman AB Prognostic Value of Usual Gait Speed in Well-Functioning Older People-Rresults from the Health, Aging and Body Composition Study. J Am Geriatr Soc (2005) 53(10):1675–80. 10.1111/j.1532-5415.2005.53501.x 16181165

[33] O’Caoimh R Sezgin D O’Donovan MR Molloy DW Clegg A Rockwood K Prevalence of Frailty in 62 Countries across the World: A Systematic Review and Meta-Analysis of Population-Level Studies. Age Ageing (2021) 50(1):96–104. 10.1093/ageing/afaa219 33068107

[34] Collard RM Boter H Schoevers RA Oude Voshaar RC. Prevalence of Frailty in Community-Dwelling Older Persons: A Systematic Review. J Am Geriatr Soc (2012) 60(8):1487–92. 10.1111/j.1532-5415.2012.04054.x 22881367

[35] Gómez JF Curcio CL Alvarado B Zunzunegui MV Guralnik J. Validity and Reliability of the Short Physical Performance Battery (SPPB): A Pilot Study on Mobility in the Colombian Andes. Colomb Med Cali Colomb (2013) 44(3):165–71. 10.25100/cm.v44i3.1181 PMC4002038 24892614

