# Supplementary material for: Short Physical Performance Battery and Study of Osteoporotic Fractures Index in the Exploration of Frailty Among Older People in Cameroon
Source: Int J Public Health. 2023 Aug 7;68:1605900. doi: 10.3389/ijph.2023.1605900 (PMC10441226; doi:10.3389/ijph.2023.1605900)
Supplement: Supplementary file 1 [file DataSheet1.pdf]

Appendix

Supplementary File 1: Short Physical Performance Battery Scale

The SPPB consists of three tests: a hierarchical assessment of standing balance, a short walk at the usual pace of older people, and the transition from sitting in a chair to standing five times [1]. For all tests, a score of zero if they were unable to do the test.

- **Balance test**

Implementation: Participants were asked to stand with their feet as close together as possible, then in a semi-tandem position (the side of the heel of one foot touches the big toe of the other foot for 10 seconds) and finally in a tandem position (the heel of one foot completely in front and touching the toes of the other foot for about 10 seconds).

Scores: The scores were assigned as follows:

- a score of 1 if they were able to stand with their feet side by side for 10 seconds, but unable to hold a semi-tandem position for 10 seconds,
- a score of 2 if they were able to hold a semi-tandem position for 10 seconds but unable to hold a full tandem position for more than 2 seconds,
- a score of 3 if they managed to stay in full tandem position for 3 to 9 seconds and
- a score of 4 if they could hold a full tandem position for 10 seconds.

- **Gait speed test**

Implementation: participants were asked to walk four meters at a normal pace and the time required to cover this distance was measured.

Scores: The scores were assigned as follows:

- a score of 1 for a duration > 8.7seconds;
- a score of 2 for a duration ranging from 6.21 to 8.7 seconds;
- a score of 3 for a duration ranging from 4.82 to 6.20 seconds; and
- a score of 4 for a duration < 4.82 seconds.

- **Chair stand test**

Implementation: Participants were asked to stand up and sit down five times as quickly as possible from a chair, keeping their arms crossed over their chest. The time taken to stand up and sit down 5 time was recorded.

Scores: The scores were assigned as follows:

- a score of 1 for a duration >16.7 seconds;
- a score of 2 for a duration ranging from 13.7 to 16.6 seconds;
- a score of 3 for a duration ranging from 11.2 to 13.6 seconds; and
- a score of 4 for a duration <11.1 seconds.

Supplementary File 2: Study of Osteoporotic Fractures index scale

Frailty as defined by the SOF index was identified by the presence of two or more of the following three components:

- Loss ≥5% of body weight (regardless of intention to lose weight).
- Inability to get up from a chair five times without using the arms,
- Reduction in energy level, identified by the participant answering “No” to the question “Do you feel full of energy?”.

The response to each component was scored, 1 if Yes and 0 if No. Higher scores indicate greater frailty.

Supplementary tables

Table S1: Sociodemographic and clinical characteristics of participants, Douala, Cameroon. 2019.

**Characteristic**	**N = 403**[1]
Age	67.0 (63.0, 71.0)
Sex (Female)	200 (49.6%)
Marital status (Living alone)	176 (43.7%)
Professionally active	163 (40.4%)
BMI (kg/m^2)	27.5 (24.3, 31.6)
Diabetes	40 (9.9%)
Hypertension	121 (30.0%)
Chronic alcoholism	30 (7.4%)
Tobacco consumption	8 (2.0%)
Cognitive impairment	207 (51.4%)
IADL score	4.0 (4.0, 4.0)
Balance test score	4.0 (3.0, 4.0)
Gait test score	3.0 (3.0, 4.0)
Chair stand test	3.0 (2.0, 4.0)
SPPB score	10.0 (8.0, 11.0)

Characteristic	N = 403[1]
SOF Index score	0.0 (0.0, 1.0)
Physical frailty	144 (35.7%)

[1] Median (IQR); n (%) ; BMI : Body mass index ; IADL : Instrumental Activity of Daily Living ; SOF: Study of Osteoporotic Fractures index; SPPB: Short Physical Performance Battery.

Table S2: Summary of diagnostic values for each threshold, Douala, Cameroon. 2019.

Threshold values	Sensitivity(%)	Specificity(%)	PPV(%)	NPV(%)	Se+Sp-1
SPPB ≤ 6	29.2	99.6	97.7	71.7	0.29
SPPB ≤ 7	52.1	96.9	90.4	78.4	0.49
SPPB ≤ 8	69.4	89.9	79.4	84.1	0.59
**SPPB ≤ 9**	**88.9**	**74.9**	**66.3**	**92.4**	**0.64**
SPPB ≤ 10	96.5	47.1	50.4	96.1	0.44

PPV: positive predictive value; NPV: negative predictive value; The best threshold is in bold

Table S3: Correlations between test components and the first two dimensions, Douala, Cameroon. 2019.

Variables	Dimension 1*	Dimension 2*
Balance	0.5529263	0.8226060
Gait speed	0.7011922	
Chair stand	0.8249393	-0.3819802
SOF	-0.8729014	
SPPB	0.9651637	
Age	-0.2485604	
Sex (R^2)	0.06725954	

* Only significant correlations are shown; SOF: Study of Osteoporotic Fractures index; SPPB: Short Physical Performance Battery.

Table S4: Projection quality in the first plane (dimensions 1 and 2), and V-test for each sex, Douala, Cameroon. 2019.

Variables	Cos2		v.test	
	Supplementary variable			
	**Dimension 1**	**Dimension 2**	**Dimension 1**	**Dimension 2**
**Female**	0.988	0.003	-5.200	-0.474
**Male**	0.988	0.003	5.200	0.474

Supplementary figures

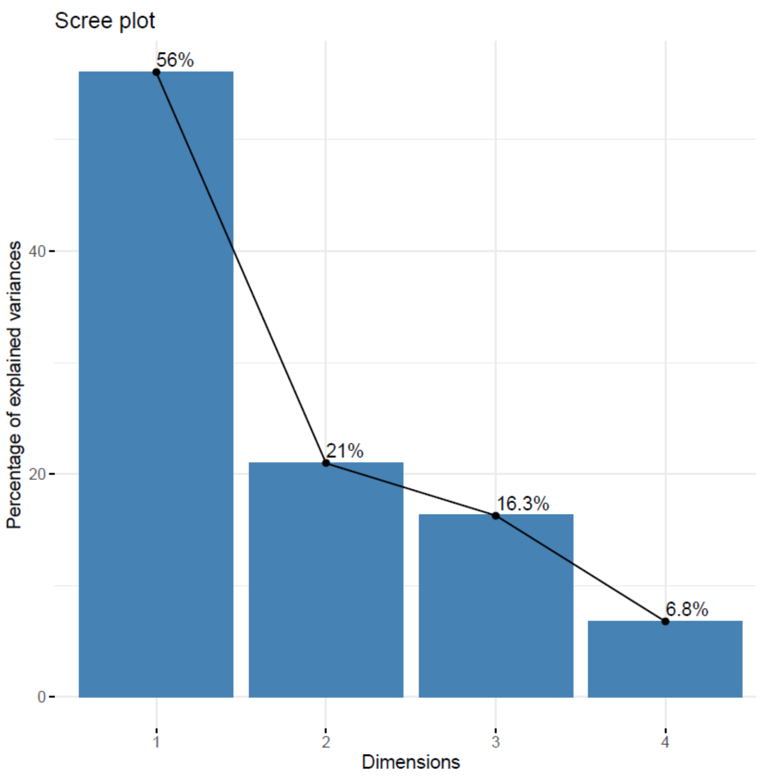

Figure S1: Percentage inertia of principal component analysis dimensions, Douala, Cameroon. 2019.

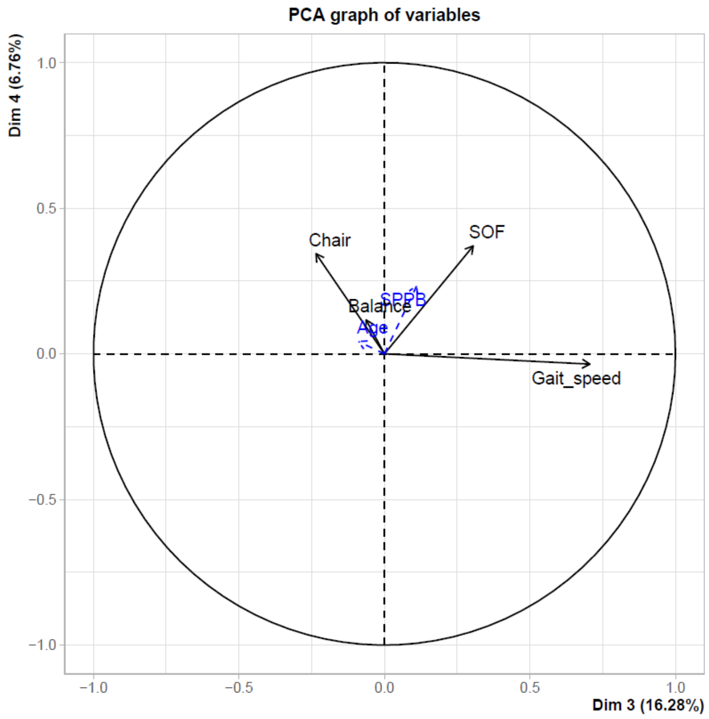

Figure S2: Representation of variables in the second plane, Douala, Cameroon. 2019.

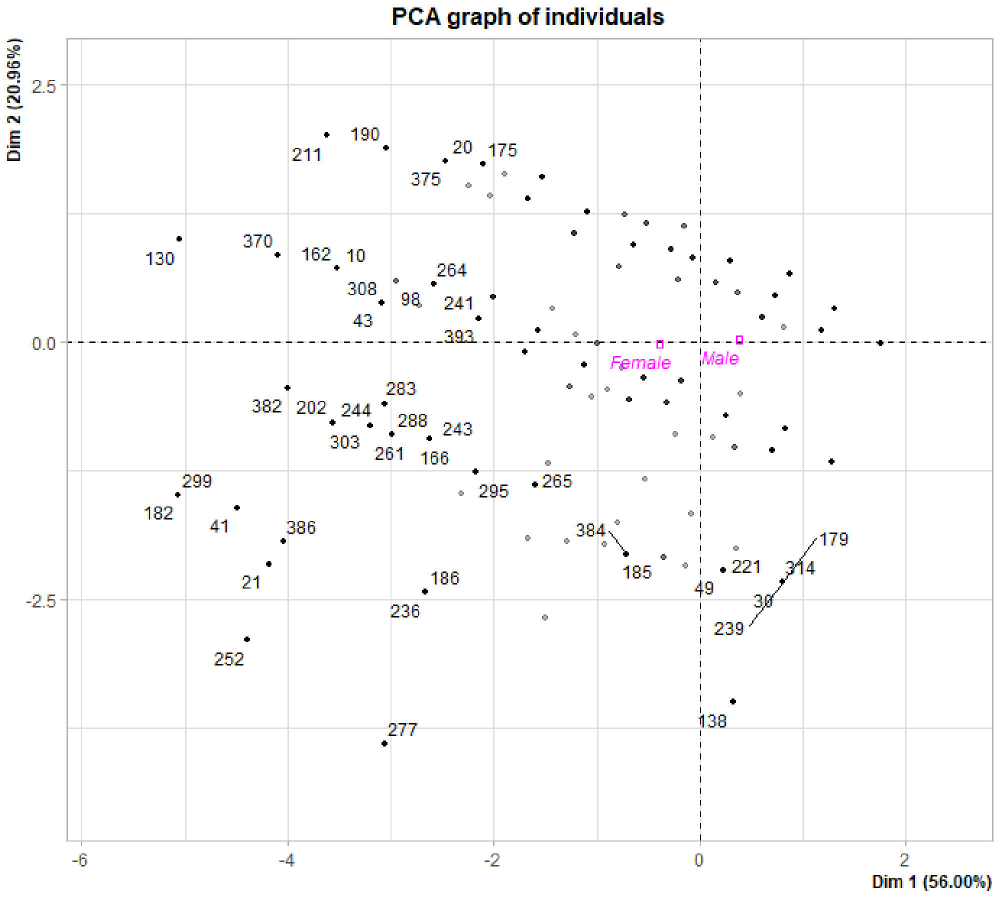

Figure S3: Principal component analysis graph of individuals, Douala, Cameroon. 2019.

References

1. Gómez JF, Curcio CL, Alvarado B, Zunzunegui MV, Guralnik J. Validity and reliability of the Short Physical Performance Battery (SPPB): a pilot study on mobility in the Colombian Andes. Colomb Medica Cali Colomb. 2013 Jul;44(3):165–71.
